# Patient-reported outcomes and functional exercise capacity in a real-life setting in non-small cell lung cancer patients undergoing stereotactic body radiotherapy: the Lung PLUS study

**DOI:** 10.3389/fonc.2023.1220248

**Published:** 2023-08-25

**Authors:** Lotte van der Weijst, Renée Bultijnck, Axel Van Damme, Vincent Huybrechts, Marc van Eijkeren, Yolande Lievens

**Affiliations:** ^1^ Department of Human Structure and Repair, Ghent University, Ghent, Belgium; ^2^ Department of Radiation Oncology, Ghent University Hospital, Ghent, Belgium

**Keywords:** health-related quality of life, non-small cell lung cancer, patient-reported outcomes, radiotherapy, toxicity

## Abstract

**Introduction:**

To better understand the impact of stereotactic body radiotherapy (SBRT) and its treatment-related toxicity on early-stage non-small cell lung cancer (ES-NSCLC) patients, we conducted the Lung PLUS study in a real-world setting.

**Methods:**

This is a monocentric prospective longitudinal study up to 12 months post-treatment, evaluating clinician- and patient-reported toxicity (resp. CTCAE and PRO-CTCAE), health-related quality of life (HRQoL) (EORTC QLQ-C30 and LC-13), activities of daily living (HAQ-DI) and functional exercise capacity (6 Minute Walking Test (6MWT)). A mixed model approach was applied to analyze the data.

**Results:**

At baseline, clinicians and patients (n=51) reported mostly fatigue (63% vs 79%), cough (49% vs 75%) and dyspnea (65% vs 73%) of any grade. Dyspnea (p=.041) increased over time. Meaningful clinical improvements were particularly seen in pain, fatigue, and cough. Clinician reported clinically meaningful improvements and deteriorations over time in fatigue, cough, and dyspnea. Almost at every timepoint, more people reported deterioration to the clinician than improvement in aforementioned toxicities. Overall HRQoL (p=.014), physical (p=.011) and emotional (p<.001) functioning improved over time. At baseline, patients had a moderate daily functioning score and walked an average distance of 360 meters. No statistically significant differences were found in daily functioning and exercise capacity over time.

**Conclusion:**

Our study showed an increase in patient-reported toxicity and dyspnea, without impacting functional status, following SBRT. Overall HRQoL, physical and emotional functioning improved over time. Understanding the impact of treatment on patient-reported outcomes is crucial to identify the needs/problems of patients to enhance their HRQoL.

## 1 Introduction

Lung cancer is the most deadly of all cancers worldwide and is only preceded in incidence by breast cancer (1). The majority of lung cancer patients (85%) are diagnosed with non-small cell lung cancer (NSCLC) of which 20% is detected at an early stage (ES) (2). The standard treatment for ES-NSCLC is surgery, but for those unwilling or unsuitable for surgery due to age, multiple comorbidities and/or poor physical function, stereotactic body radiotherapy (SBRT) is the standard of care (3). However, the unadjusted overall survival rates of SBRT at 1, 3 and 5 years are 83%, 57% and 41% respectively, which is lower than those historically observed in surgical candidates (2).

The poor survival rates of ES-NSCLC patients who are medically inoperable and treated with SBRT can be attributed to the fact that this patient population is often older, has multiple severe co-morbidities and a low performance status, as reported in previous studies (4). Although population-based and randomized controlled studies have proven the benefit of SBRT, the extent of this benefit in frail patients is frequently questioned (5, 6). Indeed, SBRT can lead to acute and late toxicities (7, 8), which can potentially affect the health-related quality of life (HRQoL) of these patients (9, 10). Yet, it is not always easy to disentangle toxicities from lung cancer symptoms, or even symptoms of intercurrent diseases, such as chronic obstructive lung disease (COPD) (8, 11). Whereas these symptoms and toxicities are usually clinician-scored, symptoms and toxicities directly reported by the patients may be different and more reliable. Hence, there is growing interest in patient-reported outcomes (PROMs). It has been recognized that the combination of PROMs and clinician-scored data provides more accurate knowledge of patient’s wellbeing (12).

HRQoL is an important measure of the impact of disease and treatment on patient’s overall wellbeing. It covers different aspects of a person’s life, including physical, psychological, social, sexual and spiritual functioning. However, HRQoL can be difficult to interpret because it is such a complex concept. HRQoL is often captured with PROMs (13).

Lastly, lung cancer and its treatments may also have a substantial impact on a patient’s functional and physical wellbeing, subsequently influencing their HRQoL (14). Lung cancer patients are particularly at risk for exercise intolerance, muscle fatigue, impaired lung functions and pulmonary complications (15). Furthermore, baseline physical wellbeing and physical exercise capacity are prognosticators for treatment response and survival in lung cancer (16). This may aid in decision-making regarding the most optimal therapy.

Ample research has been conducted on PROMs in ES-NSCLC patients receiving SBRT (17–25). However, none of these studies collected comprehensive data on patient-reported HRQoL, symptoms, toxicity and daily living activities along with clinician-scored toxicity data, nor were data collected in a real-world setting. Real-world evidence provides inclusive data on the heterogeneous lung cancer population. As patients receiving SBRT are characterized by poor performance status and co-morbidities, these patients are frequently excluded from clinical trials (26). The REQUITE project included a large group of ES-NSCLC patients treated with SBRT in the real-world setting, but did not collect patient-reported toxicities (9, 10). Therefore, there is a need for further research that collects comprehensive patient-reported data in a real-world setting to improve our understanding of the impact of SBRT on ES-NSCLC patients.

The Lung PLUS study is a real-life, prospective, longitudinal study that investigated PROMs (symptoms, toxicities, HRQoL and activities of daily living) along with clinician-scored symptoms and toxicity, and functional exercise capacity in ES-NSCLC patients receiving SBRT. Hereby, we present both baseline and longitudinal data related to symptoms, toxicity, HRQoL, and activities of daily living. Additionally, we compare the symptoms/toxicities scored by patients and clinicians and examine whether HRQoL and physical functioning have an impact on survival.

## 2 Materials and methods

### 2.1 Patient population and treatment

For this monocentric prospective, longitudinal cohort study, we recruited ES-NSCLC patients without any other malignancies in the 5 years leading up to the NSCLC diagnosis. Additionally, we only included patients with an Eastern Cooperative Oncology Group (ECOG)/World Health Organisation (WHO) performance status of ≤2, who underwent SBRT at Ghent University Hospital (GUH) in Belgium. The study was approved by GUH’s ethical committee (EC 2017/0517), and all patients provided written informed consent before enrolling in the study.

Data concerning patient and tumor characteristics were collected at baseline, whereas details regarding SBRT were obtained at the end of radiotherapy.

### 2.2 Outcome measures

Patient-reported symptom and toxicity data on pain, fatigue, dyspnea, cough and dysphagia were collected with the Patient-Reported Outcomes Version of the Common Terminology Criteria for Adverse Events (PRO-CTCAE) (27).

Clinicians scored lung cancer-related symptoms (fatigue, cough and dyspnea) and radiotherapy-induced toxicities (dysphagia, esophagitis, hemoptysis, chest wall pain, pneumonitis and radiotherapy-dermatitis) with the Common Terminology Criteria for Adverse Events (CTCAE) (28).

The patient-reported and clinician-scored data collected during the baseline assessment were categorized as symptom data, while the data obtained at subsequent time points were classified as toxicity data (29).

HRQoL data was collected with the European Organisation for Research and Treatment of Cancer (EORTC) Quality of Life Questionnaire Core 30 items (QLQ-C30) and the EORTC Quality of Life Questionnaire Lung Cancer 13 items (QLQ-LC13) (30–32). The QLQ-C30 evaluates five functional domains (physical, role, emotional, cognitive, and social), nine cancer-related symptoms, global health and quality of life. The QLQ-LC13 measures lung cancer-specific symptoms.

Data was collected from both patient-reported and clinician-scored sources prior to the initiation of radiotherapy, as well as at 1, 3, and 12 months following SBRT treatment. These specific time points were chosen as they align with the follow-up consultations that patients receive at Ghent University Hospital (GUH). Additionally, patient-reported HRQoL, functional status and toxicity data was collected at 6 and 9 months post-treatment. Functional exercise capacity was measured before the start of radiotherapy, and at 3 and 12 months with the six-minute walk test (6MWT). Participants were asked to walk self-paced for six minutes on a hard flat straight surface. The 6MWT was performed according to previously published recommendations (33).

Daily functioning was measured using the self-administered health assessment questionnaire disability index (HAQ-DI). This questionnaire evaluates activities of daily living over the past week across eight categories, including dressing and grooming, arising, eating, walking, hygiene, reaching, gripping and errands and chores. Data on specific aids, devices utilized for assistance or whether help was needed from another person were captured as well (34). Survival data, defined as the time between study inclusion and death as a result of any cause, was additionally collected. Two-year survival data was available for all patients.

### 2.3 Data handling and statistical analysis

Patient- and clinician-scored toxicities were calculated by subtracting baseline scores from follow-up scores.

HRQoL data of the EORTC QLQ-C30 and LC13 was calculated following the guidelines of the EORTC (31). Functional scales, global quality of life, and health status scores that are higher in value indicate better functioning. Conversely, symptom scales that have higher scores indicate a greater presence of symptoms. HRQoL data was regarded as missing if at least half of the items were missing from the EORTC QLQ-C30 and LC13 questionnaires. In the analyses, only data on the different domains and overall HRQoL was included. For the purposes of this study, a meaningful clinical important difference (MCID) was defined as a score difference of at least 10 points within a patient between 2 different time points (35).

A MCID in functional capacity was defined as any change in walking distance that exceeded the initial distance by 9.5% (36). Daily functioning status of the HAQ-DI was calculated using following the guidelines of the questionnaire (37). Data was considered missing if more than two of the eight categories were missing. The total score is between 0 and 3.0, in 0.125 increments. An increase in score indicates worsening of functioning, with 0 indicating no functional impairment and 3 indicating complete impairment. Scores of 0 to 1 are considered to represent mild to moderate difficulty, 1 to 2 moderate to severe disability, and 2 to 3 severe to very severe disability. A minimal clinically important difference was defined as 0.22 (38).

For patient, treatment, and tumor characteristics, as well as for changes from baseline, descriptive statistics were utilized. To determine statistical significance levels, the mixed model method was employed with a compound symmetry structure (39). This analytical technique was selected due to its ability to handle hierarchical and missing data, as well as repeated measurements from individual patients. A statistical significance level of p=0.05 was established to adjust for both multiple comparisons and to account for the risk of a level I error. Finally, exploratory survival analyses were conducted (based on the median) to evaluate the impact of baseline data on survival.

## 3 Results

### 3.1 Patient and treatment characteristics

The Lung PLUS study enrolled 51 patients between June 2017 and December 2020. There was a male predominance (n=37; 73%) and most patients had an Eastern Cooperative Oncology Group (ECOG)/World Health Organisation (WHO) performance status of 1 (n=25; 49%). Furthermore, 35 patients (69%) had cardiovascular co-morbidities and 34 individuals (67%) had pulmonary co-morbidities. The majority of patients presented cT1bN0M0 (n=26; 51%) disease and the most commonly used fractionation schemes were 60 Gy in 3 fractions (n=18; 35%) and 60 Gy in 8 fractions (n=26; 51%). **Table 1** provides an overview of patient, tumor and SBRT characteristics. **Table 2** provides details on baseline HRQoL.

**Table 1:** Patient, tumor and treatment characteristics.

Patient characteristics	N (%) or mean (range)
Male	37 (73)
Age at study inclusion	72 (52–87)
WHO Performance Status	
0	11 (22)
1	25 (49)
2	15 (29)
Comorbidities	
Cardio-vascular disease	35 (69)
Hypertension	25 (49)
Other cardio-vascular disease	25 (49)
Pulmonary disease	34 (67)
Asthma	7 (14)
COPD	31 (62)
Diabetes	6 (12)
Previous malignancies*	11 (22)
BMI	
Underweight (<18.5)	5 (10)
Normal (18.5 – 24.9)	30 (59)
Overweight (25 – 29.9)	11 (22)
Obese (>30)	4 (8)
Missing	1 (2)
Smoking status	
Never smoker	4 (8)
Ex-smoker before cancer diagnosis	18 (35)
Ex-smoker, since cancer diagnosis	7 (14)
Current	22 (43)
Alcohol use	
No	15 (29)
Quit before cancer diagnosis	3 (6)
Quit since cancer diagnosis	2 (4)
Current use	28 (55)
Unknown	3 (6)
Highest education	
Primary school	21 (41)
Secondary school	22 (43)
University	8 (16)
Tumor characteristics	N (%)
TNM classification	
cT1aN0M0	4 (8)
cT1bN0M0	26 (51)
cT1cN0M0	14 (28)
cT2aN0M0	4 (8)
cT2bN0M0	1 (2)
cT3N0M0	1 (2)
cT1bNxM0	1 (2)
Histology	
Adenocarcinoma	7 (14)
Squamous-cell carcinoma	7 (14)
No pathology available	37 (72)
Treatment characteristics	
SBRT scheme	
3x20Gy	18 (35)
4x15Gy	1 (2)
5x12Gy	4 (8)
8x7.5Gy	26 (51)
No radiotherapy given**	2 (4)

*Previous malignancies were prostate cancer, head and neck cancer, breast cancer, colon cancer, lymphoma, esophageal cancer, lung

**Radiotherapy was not given due to death before start of radiotherapy (n=1) or change of treatment plan (n=1).

WHO, World Health Organisation performance status; SBRT, stereotactic body radiotherapy.

**Table 2:** Health-related quality of life characteristics.

EORTC QLQ-C30 scores	Average score (Q1-Q3)
Physical functioning	60.7 (33 – 67)
Role functioning	66.0 (33 – 66)
Emotional functioning	67.3 (33 – 77)
Cognitive functioning	77.9 (33 – 83)
Social functioning	79.6 (33 – 100)
Fatigue	33.6 (0 - 58.3)
Nausea and vomiting	4.1 (0 - 0)
Pain	25.5 (0 - 37.5)
Dyspnoea	42.9 (25 - 50)
Insomnia	34.7 (0 - 50)
Appetite loss	13.6 (0 - 25)
Constipation	11.7 (0 - 0)
Diarrhea	6.1 (0 - 0)
Financial difficulties	11.7 (0 - 25)
Overall HRQoL	75.3 (38.8 – 85)
EORTC QLQ-LC13 scores	
Cough	38.8 (25 - 37.5)
Haemoptysis	2.8 (0 - 0)
Dyspnoea	36.0 (25 - 50)
Sore mouth	12.4 (0 - 0)
Dysphagia	8.2 (0 - 18.8)
Peripheral neuropathy	15.3 (0 - 18.8)
Alopecia	4.9 (0 - 0)
Pain in chest	10.9 (0 - 12.5)
Pain in arm or shoulder	18.7 (0 - 25)
Pain in other parts	28.4 (0 - 50)

Interpretation scores; higher scores in the functional scales, global quality of life and health status stipulate better functioning. Higher scores in the symptom scales indicate more symptoms.

HRQoL, health-related quality of life.

### 3.2 Compliance

The overall compliance rate for PROMs was 96% (n=49) at baseline, and 95% (n=42), 89% (n=40) and 67% (n=29) at 1-, 3- and 12-months post-radiotherapy respectively. The compliance rate with the 6MWT was substantially lower, with only 69% (n=35) of patients performing the test at baseline, 66% (n=29) at 3 months and 45% (n=20) at 12 months. The reasons for this lower compliance rate are diverse and are reported in **Table 3** which provides a synopsis of the number of data collected and compliances to the study.

**Table 3:** Data availability.

Time point	Available data n (%)				
	Patient-reported toxicity	Clinician-reported toxicity	HRQoL	HAQ-DI	6MWT*
Baseline	49 (96)	50 (98)	49 (96)	49 (96)	35 (69)
1 month	41 (93)	44 (100)	42 (95)	40 (91)	–
3 months	40 (89)	44 (100)	40 (89)	40 (89)	29 (66)
6 months	31 (70)	–	32 (73)	31 (70)	–
9 months	25 (57)	–	25 (57)	26 (59)	–
12 months	28 (65)	40 (91)	29 (67)	29 (66)	20 (45)

Clinician-reported toxicity was not collected per protocol at month 6 and 9. 6MWT was not collected per protocol at month 1, 6 and 9.

6MWT, 6-minute walk test; HAQ-DI, health assessment questionnaire disability indiex; HRQoL, health-related quality of life.

*reasons for not administring the 6MWT were; continuous oxygen therapy, visually impaired, patient refusal, pain, general weakness, leg amputation, wheelchair, COVID-19 (telephone consultation) or lost to follow-up.

### 3.3 Patient-reported symptoms and toxicity

**Figure 1:**
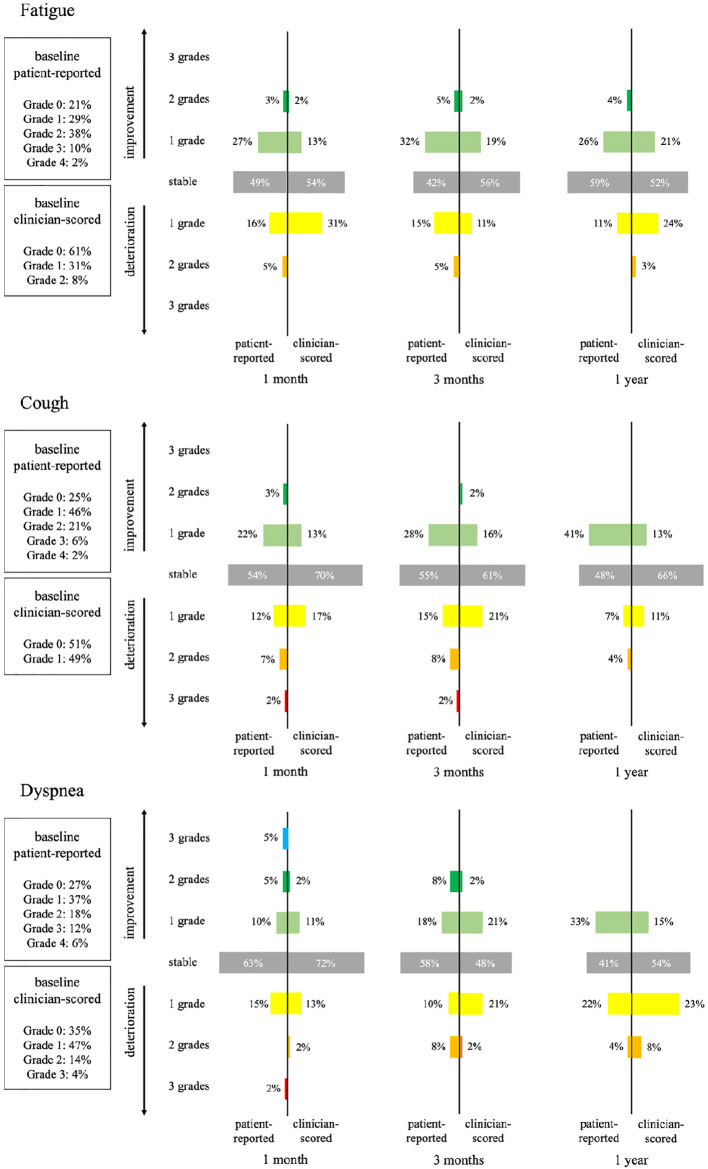
Comparison of patient-reported and clinician-scored toxicity: fatigue, cough and dyspnea.

At baseline, many patients reported pain (57%), fatigue (79%), cough (75%) and dyspnea (73%) of at least grade one. Baseline dysphagia was the least reported (24%). See **Figure 1**. for more details. Dyspnea (p=0.041) significantly increased over time, whereas pain (p=0.087), fatigue (p=0.275), cough (p=0.175) and dysphagia (p=0.641) remained stable. In terms of MCIDs, particularly pain, fatigue and cough improved significantly over time. At the 1 month post-treatment mark, dyspnea showed greater deterioration in comparison to other areas of improvement. However, at both the 3 and 12 month post-treatment marks, more improvement than deterioration was observed. Dysphagia, on the other hand, was not frequently reported and generally showed improvement by the 12 month mark.

### 3.4 Clinician-reported symptoms and toxicity

At baseline, clinicians noted mostly fatigue (63%), cough (49%) and dyspnea (65%). The other symptoms were rarely reported. An overview of the comparison of patient-reported and clinician-scored symptoms and toxicities for fatigue, cough and dyspnea can be found in **Figure 1**. Clinicians reported fewer symptoms/toxicity than patients.

With regards to clinical significance, fatigue, cough, and dyspnea showed both improvement and deterioration over time. At nearly every time point, more clinicians reported deterioration than improvement in these toxicities. At three months, there were no patients with an increase in dyspnea after SBRT that had progressive disease. At 12 months 15% (2 out of 13) in the patients with an increase of dyspnea after SBRT had progressive disease.

Although chest wall pain and pneumonitis were not commonly reported at baseline, a small percentage of patients experienced these toxicities after receiving radiotherapy. Radiation dermatitis was observed in a relatively small number of patients and occurred particularly at the 1 month post-treatment mark.

### 3.5 Health-related quality of life

At baseline, the average overall score of HRQoL was 75. The poorest score was reported for physical (61) and role (66) functioning. The most severe symptoms were dyspnea (43/36 on the QLQ-C30 and LC13 respectively) and cough (39) and insomnia (35) on the QLQ-LC13 module.

**Figure 2:**
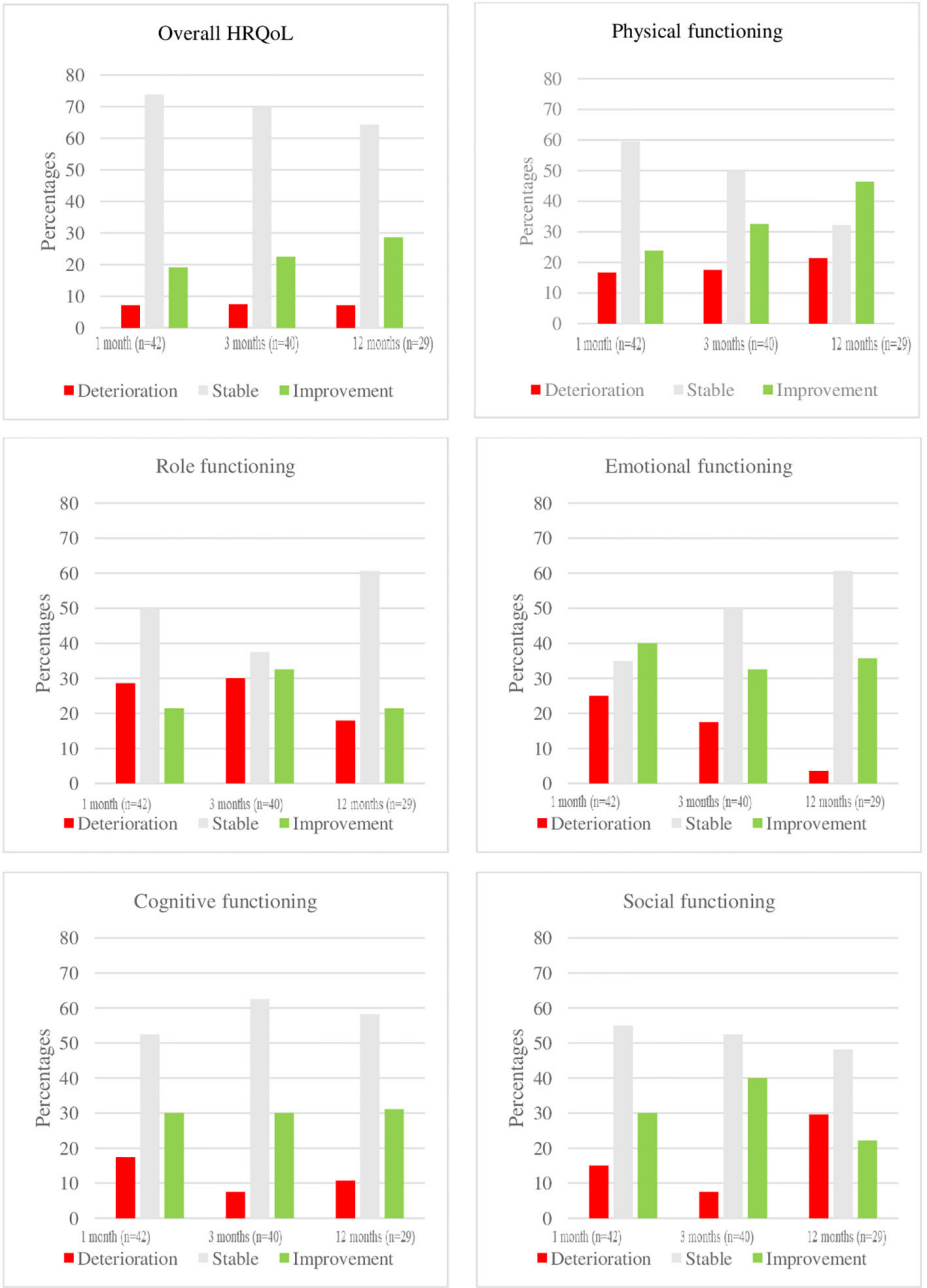
Overview of MCID of HRQoL and its domains per time point. A meaningful clinical important difference (MCID) was defined as a score difference of at least 10 points within a patient between 2 different time points.

Overall HRQoL (p=0.014), physical (p=0.011) and emotional (p<.001) functioning improved significantly over time, whereas the other domains (role (p=.606), cognitive (p=.076) and social functioning (p=0.570) remained stable. When considering MCID, improvements over time were primarily observed in overall HRQoL and physical functioning, while the deterioration of emotional functioning decreased over time. For an overview of MCID for overall HRQoL and its associated domains, please refer to **Figure 2**.

### 3.6 Functional exercise capacity

**Figure 3:**
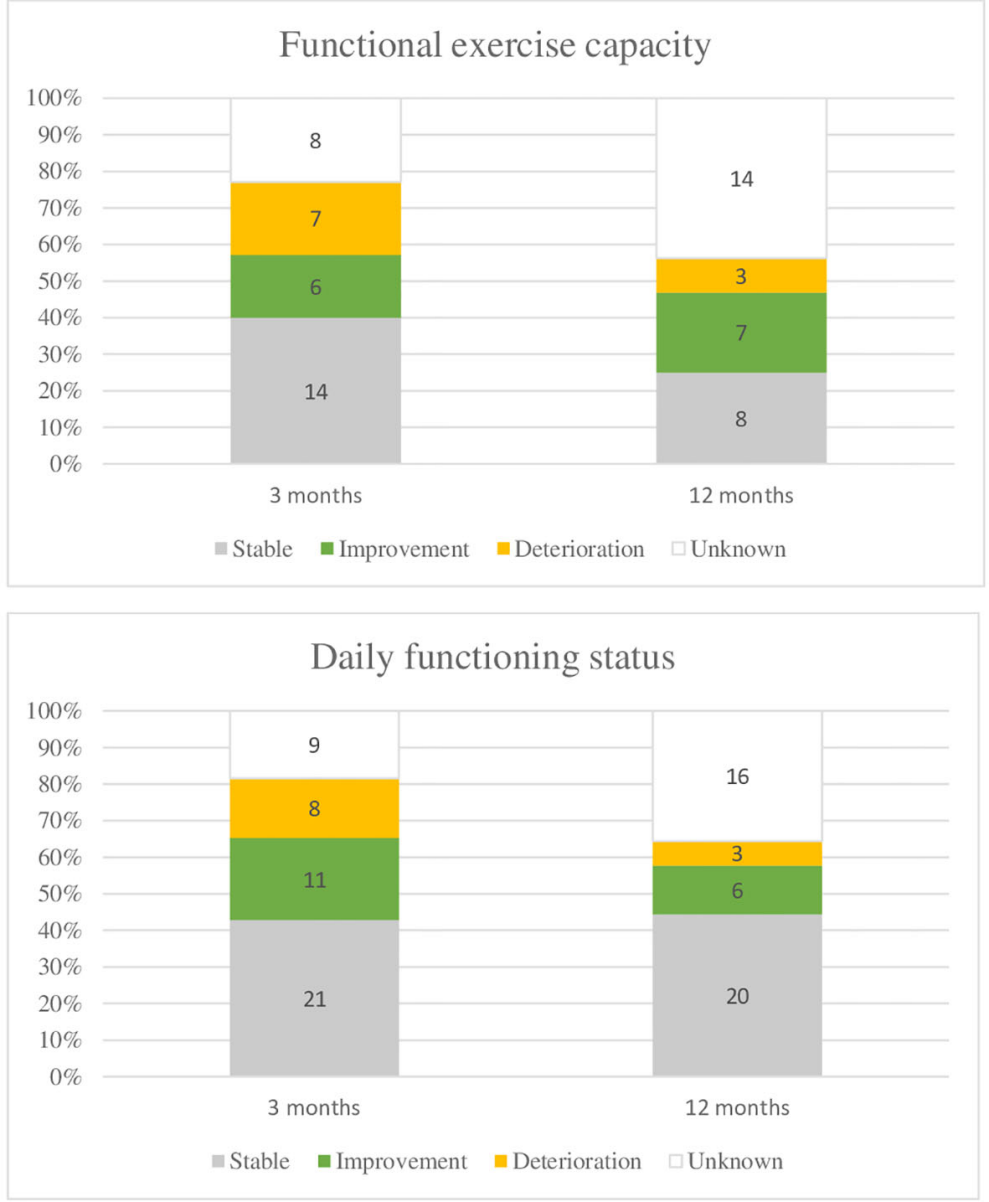
Evolution of functional exercise capacity and daily functioning, at 3 months and 12 months. Functional exercise capacity was measured with the six-minute walk test (6MWT), daily functioning with the health assessment questionnaire disability index (HAQ-DI). At three months, 2 patients died and at 1 year. 5 patients died. In addition, 15 resp. 14 patients were excluded from the 6MWT for a number of reasons. Lost to follow up or administrative failure was reported as unknown. The minimal clinical important difference was determined based on a 9.5% change from baseline for the 6MWT (36); for the daily functioning status, it was determined at 0.22 (38).

At baseline, patients had an average walking distance of 359.1 meters (±107, IQR 317-431). The distance remained relatively stable at 3 months (mean 351.1 ± 115 meters, IQR 290-412) and at 12 months (mean 350.9 ± 105 meters, IQR 287-416), with no statistically significant differences observed over time (p=0.862). **Figure 3** provides an overview of MCID. No adverse events or medical conditions were observed during the tests. Four patients stopped prematurely or took a break during the test, 2 at the baseline test, one at month 3 and one at month 12.

### 3.7 Daily functioning

At baseline, an average score of daily functioning, measured with the HAQ-ID was 0.813, (IQR 0.062-1.500), while it was 0.812 (IQR 0.125-1.458) and 0.655 (IQR 0-1.312) at 3 and 12 months respectively. No statistically significant difference (p=0.435) was found in daily functioning over time. An overview of MCIDs can be found in **Figure 3**.

### 3.8 Survival

**Figure 4:**
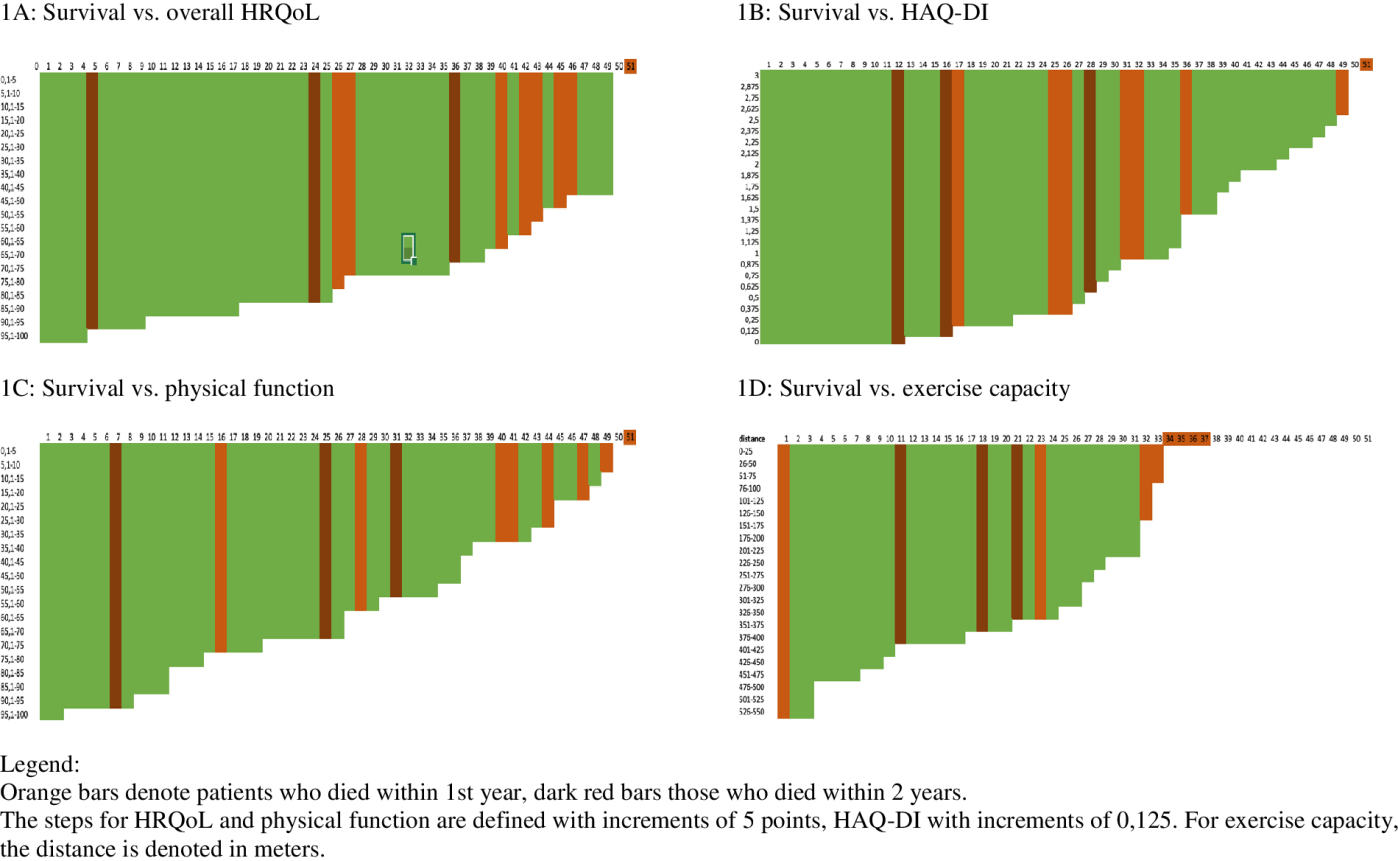
Survival in function of baseline overall HRQoL, physical and daily functioning and exercise capacity. x-as patient ID; y-as PRO scoring or exercise capacity score.

Our exploratory analyses did not show any significant results for baseline overall HRQoL (p=0.068), physical functioning (p=0.079), daily functioning (p=0.261) and exercise capacity (p=0.062). Our findings suggest that patients who reported higher baseline scores for overall HRQoL, physical and daily functioning, as well as exercise capacity, were more likely to have a favorable 1-year survival prognosis. We present exploratory survival plots in **Figure 4** to further illustrate these associations.

## 4 Discussion

This study aimed to assess the symptom and toxicity scores reported by patients and clinicians, as well as patient-reported HRQoL and its associated domains among ES-NSCLC patients undergoing SBRT. This group of patients is considered vulnerable due to their poor overall and physical health, multiple co-morbidities, and high symptom burden. Thus, it is crucial to comprehend the symptoms associated with the disease, the toxicities induced by radiotherapy, and their influence on decision-making related to HRQoL.

In this study, many patients reported pre-treatment symptoms, particularly fatigue, cough and dyspnea. Fatigue is the most common symptom experienced in patients with cancer, lung and cardio-vascular disease (40, 41). Cancer-related fatigue affects approximately 65% of patients after treatment (41). Cough and dyspnea are typical symptoms of COPD, a condition present in the majority of our patients (n=31; 61%) (42). There was a significant increase in dyspnea over time. Previous studies have also reported a deterioration in dyspnea over time in ES-NSCLC patients who underwent SBRT, which is consistent with our findings (43). Dyspnea correlates with multiple medical factors, co-morbidities, tumor growth. and psychological factors, such as anxiety and depression.

On the other hand, pain, fatigue, and cough that were predominantly present before the treatment showed improvements over time. Our study observed improvements in patient-reported overall HRQoL, physical functioning, and emotional functioning. This is consistent with a previous study (n=39) in a similar patient population, which found a significant improvement in emotional functioning (p=0.002). However, that study reported that overall HRQoL, physical functioning, and respiratory symptoms remained stable. It is possible that the improvement in emotional well-being is linked to decreased anxiety and depression during follow-up (21). In contrast, Schwartz et al. (20) (n=28) found that HRQoL deteriorated after treatment, particularly the physical and mental health. The deterioration in HRQoL in those receiving SBRT was comparable to ES-NSCLC patients receiving surgery. Patients referred to SBRT for ES-NSCLC, as typical of this patient population, had a notably poorer physical and mental functioning before treatment.

Rutkowski and colleagues, using the EORTC QLQ-C30 and lung cancer module (QLQ-LC13), and concluded that physical (p=.032) and emotional functioning (p<.001) wellbeing improved significantly and clinically meaningful at 3 months in those (n=51) receiving SBRT (21). However, most improvements were seen in patients without COPD.

In terms of MCIDs in HRQoL, REQUITE, a large international cohort study, showed different results (9, 10). Whereas in our study, more patients improved in overall HRQoL, its associated domains and all functional domains over time, in the REQUITE study more patients clinically meaningfully deteriorated in overall HRQoL. One possible explanation for this is the variation in the significance placed on different aspects of HRQoL across different patient populations and cross-cultural (44).

The findings indicate that a larger proportion of patients exhibited gradual improvements in overall HRQoL and its related domains compared to deterioration. It should be noted that patients with better baseline health and HRQoL tend to have a more favorable prognosis and are less likely to drop out of the study. Therefore, the results of this study may primarily apply to individuals with higher baseline performance status and overall health.

The results of the 6MWT, which assesses functional exercise capacity, confirm the vulnerability of our patient population. The average walking distance at baseline was 359 meters. Previous research in a similar population (n=306, inoperable NSCLC patients) has also shown comparable results, with an average of 307 meters at baseline (45). However, these results are lower compared to pre-operative lung cancer patients (n=50) who are eligible for surgery (e.g., an average of 477 meters, IQR 417-536) (46) or other tumor groups (an average of 594 meters, as seen in a study of n=50, both curative and palliative breast and colorectal cancer) (47). Previous research has shown the potential predictive value of this simple, safe, and inexpensive 6MWT on survival. They have demonstrated a cut-off distance of 525 meters to distinguish between the group of lung cancer patients eligible for surgery with better long-term survival and those with worse long-term survival (16). In our cohort, only 3 patients had a walking distance above this threshold, which further highlights the vulnerability of our population compared to those suitable for surgery. Additionally, our exploratory survival analyses did not show significant results, but suggest that patients with a low walking distance on the 6MWT or those unable to perform the test may have a worse survival. Therefore, future research should investigate the potential predictive value of the 6MWT and determine the corresponding threshold for specifically ES-NSCLC receiving SBRT.

PROMs were used to collect symptom, toxicity and HRQoL data. The advantages of PROMs are well-known (48, 49). A discrepancy between patient- and clinician-scored symptoms/toxicities has been noted, particularly regarding symptomatic toxicities such as fatigue (12), and PROMs seem to detect potentially serious symptoms earlier than clinician reporting (50). Thus, both patient and clinician-scored, as collected in this study, data are important to provide a more accurate understanding of patient’s underlying health status and functional status.

We noted discrepancies between patient and clinician-reported outcomes with the largest difference in cough (75% vs 49% for patients vs clinicians respectively) at baseline. It is known that clinicians may underreport symptoms, particularly the more subjective symptoms such as fatigue (12).

This study evaluated both statistical significance levels and MCID to determine the changes in HRQoL over time. Statistical significance levels evaluate the reliability of the data and estimate the probability that the differences in the observed size could be because of a sampling error (51). Statistical significance lacks capture of the clinical importance of the data. MCID refers to what patients perceive as beneficial and would mandate a change in patient management. As data can be statistically non-significant due to insufficient power, MCID data can provide the real-world effects of an intervention and whether it is perceived as beneficial by the patient (52).

To distinguish between pre-existing symptoms and treatment-induced toxicity. Regarding toxicity data, changes over time were calculated for toxicity data by subtracting the baseline data from subsequent data. This differentiation is important as certain symptoms related to the tumor and co-morbidities may be alleviated by treatment, whereas other toxicities may emerge due to treatment. As pulmonary symptoms are commonly reported in lung cancer patients, it is crucial to understand the impact of treatment in alleviating symptoms to facilitate informed decision-making in this vulnerable patient population.

Data was collected in a real-world setting. Real-world evidence refers to data routinely collected from daily clinical practice. As randomized-controlled trials apply strict inclusion and exclusion criteria, often excluding patients with poor performance status and multiple co-morbidities, real-world studies provide comprehensive data on a larger patient population. This study aimed to provide data on ES-NSCLC patients, a group that is characterized by poor performance status, overall health, and a multitude of pulmonary and cardiovascular co-morbidities and is therefore often excluded from clinical trials. The aim was to collect data on the standard treatment for ES-NSCLC patients ineligible or unwilling to undergo surgery and to provide evidence applicable to most of this patient population.

Future research should focus on obtaining additional real-world data in this heterogeneous patient population, as this is rather an explorative study due to the small sample size. Prospective real-world data is needed to complement safety and efficacy data from clinical trials. The introduction of new treatments, such as immunotherapy, in this patient cohorts calls for more clinical trial and real-world data. Currently, no HRQoL and patient-reported toxicity data is available in ES-NSCLC patients receiving SBRT and immunotherapy in daily clinical practice. Data collection was difficult, due to the COVID-19 pandemic and to patients’ health deterioration and death. More research is needed to ensure high completion rates in this vulnerable populations and electronic data collection possibilities should be explored.

There are several limitations to this study. Firstly, recruitment was slow due to the introduction of new studies, particularly those involving immunotherapy. Secondly, missing data was a problem. Due to the emergence of COVID-19, consultations were frequently conducted over the phone, which increased the likelihood of missing data since patients were asked to fill out PROMs through the post. Additionally, missing data occurred due to patients’ health deterioration and death.

The results from this study are mostly applicable to those with better pre- and post-treatment health. The small sample size and the data collection from only one hospital may limit the generalizability of the findings.

## 5 Conclusion

To summarize, this study found that dyspnea increased over time while certain pre-existing symptoms improved and new toxicities emerged following SBRT. Clinicians mostly reported fatigue, cough, and dyspnea, and reported less toxicity than patients. Patient-reported overall HRQoL, physical, and emotional functioning significantly improved over time, with some patients experiencing meaningful improvements and deteriorations in these domains. Results of the 6MWT remained stable over time but were relatively lower compared to other treatment and tumor groups for those able and willing to conduct the test. ES-NSCLC patients ineligible for surgery are typically older with multiple co-morbidities and poor performance status, so it is important to consider the impact of treatment on symptoms, toxicities, functioning, and HRQoL in making treatment decisions for this vulnerable population. However, this study had limitations, such as slow recruitment and missing data due to COVID-19 and patient deterioration. The data may be more applicable to those with better pre- and post-treatment health, and the small sample size and single hospital data collection further limit generalizability.

## Data availability statement

The authors confirm that the data supporting the findings of this study are available within the article and its supplementary materials. Raw data were generated at Ghent University Hospital. The raw data are not publicly available due to privacy and ethics restrictions. Raw data supporting the findings of this study are available from the corresponding author [RB] upon reasonable request.

## Ethics statement

The studies involving human participants were reviewed and approved by Commissie voor medische ethiek | UZ Gent (2017/0517). The patients/participants provided their written informed consent to participate in this study.

## Author contributions

Conceptualization: RB, LW, and YL. Methodology: RB, LW, and YL. Formal analysis: RB and LW. Data curation: RB and LW. Investigation: all authors. Project administration: RB and LW. Resources: RB and LW. Validation: RB, LW, and YL. Visualization: RB, LW, and YL. Writing – original draft preparation: RB, LW, and YL. Writing – review & editing: VH, AV, and ME. Supervision: LY. All authors contributed to the article and approved the submitted version.
